# Higher fibrinogen levels contributes to thrombosis in intracranial atherosclerosis-related large vessel occlusion strokes

**DOI:** 10.3389/fneur.2026.1753192

**Published:** 2026-03-04

**Authors:** Yimeng Liu, Hongchen Zhao, Yifeng Ling, Fangzhe Chen, Zigao Wang, Yiting Mao, Qiang Dong, Wenjie Cao

**Affiliations:** 1 Department of Neurology, Huashan Hospital, Fudan University, Shanghai, China; 2 State Key Laboratory of Medical Neurobiology, Fudan University, Shanghai, China; 3 National Clinical Research Center for Aging and Medicine, Huashan Hospital, Fudan University, Shanghai, China

**Keywords:** fibrinogen, intracranial atherosclerotic stenosis, large-vessel occlusion stroke, pathogenesis, thrombosis

## Abstract

**Background:**

The mechanisms underlying large-vessel occlusion strokes (LVOS) caused by intracranial atherosclerosis (ICAS) remain incompletely understood. This study aimed to characterize the distinct features of ICAS-LVOS to elucidate its pathological basis.

**Methods:**

We conducted a cross-sectional analysis of a prospective, single-center cohort of acute ischemic stroke patients (January 2017 to January 2023). Participants were classified into three groups: ICAS-LVOS, atrial fibrillation-related LVOS (AF-LVOS), and symptomatic ICAS without LVOS (sICAS). Clinical and laboratory data were compared.

**Results:**

The study included 279 patients, comprising 70 ICAS-LVOS patients, 78 AF-LVOS patients, and 131 sICAS patients. Compared to AF-LVOS, ICAS-LVOS patients demonstrated associations with younger age (OR: 0.898; *p* = 0.007), previous stroke (OR: 6.672; *p* = 0.031), posterior circulation involvement (OR: 30.299; *p* = 0.011) and higher fibrinogen levels (OR: 3.421; *p* = 0.006). A ratio of fibrinogen/D-dimer ≥6 effectively identified ICAS-LVOS with high specificity. Relative to sICAS, ICAS-LVOS was associated with higher body mass index (OR: 1.176; *p* = 0.002), white blood cell counts (OR: 1.234; *p* = 0.002), and fibrinogen levels (OR: 1.600; *p* = 0.029). Within the ICAS-LVOS group, higher thrombus burden was correlated with hypertension (OR: 6.071; *p* = 0.029) and higher fibrinogen levels (OR: 2.322; *p* = 0.046). Notably, in patients with fibrinogen levels <3.2 g/L, intravenous thrombolysis was associated with fewer passes of thrombectomy devices.

**Conclusion:**

ICAS-LVOS exhibits a unique profile distinct from AF-LVOS and sICAS. Fibrinogen appears to play a significant role in thrombogenesis and the occurrence of LVOS in ICAS, influencing thrombus characteristics and potentially modifying the efficacy of thrombolysis in specific patient subgroups.

## Highlights

- *What is already known*: The specific mechanisms by which intracranial atherosclerosis (ICAS) leads to large-vessel occlusion stroke (LVOS) remain poorly defined, unlike the well-established causes of cardioembolic stroke.
- *What this study adds*: We identify that ICAS-LVOS has a distinct profile, including younger age and higher fibrinogen levels. A fibrinogen/D-dimer ratio ≥6 helps identify it, and fibrinogen is linked to both its occurrence and a heavier thrombus burden.
- *How this study might affect research, practice or policy*: Fibrinogen could be a future therapeutic target, and the fibrinogen/D-dimer ratio may aid in early etiology diagnosis, potentially guiding acute treatment decisions.

## Introduction

Intracranial atherosclerotic stenosis (ICAS) is one of the most common causes of acute ischemic stroke worldwide, especially in Asian populations, with high recurrent risks (1). The rise in mechanical thrombectomy (MT) for large-vessel occlusion stroke (LVOS) has revealed ICAS as an underlying etiology in approximately 27.7% of cases (2), reaching up to 60% in Chinese populations (3). ICAS-LVOS often leads to larger infarcts, higher disability rates, and increased mortalities. Diagnosing ICAS-related LVOS poses unique challenges, relying on complicating identification during MT procedures in LVOS populations (4). Thus, studies focusing on ICAS-LVOS are scant and demanding. The hypothesis of the mechanism behind ICAS-LVOS was akin to ST-segment-elevation myocardial infarction in cardiac scenarios (5), suggesting that the acute plaque rupture leads to thrombosis and occlusion. Furthermore, given residual stenosis and compromised perfusion, over one-third ICAS-LVOS patients experience intraprocedural reocclusion, 20 times higher than non-ICAS-LVOS cases (2). Achieving and sustaining recanalization often necessitates rescue interventions such as angioplasty, stenting and intra-arterial antiplatelet administration. However, the benefit of intravenous thrombolysis (IVT) remains unclear. Hence, our study aims to identify key factors contributing to or exacerbating ICAS occlusion, shedding light on the mechanism of ICAS- LVOS and exploring more effective therapies.

## Methods

### Study design and population

A prospective cohort study of AIS patients admitted to hospital within 24 h of symptom onset was conducted at our institution. We screened 989 patients with comprehensive clinical data and angiography (digital subtraction angiography, DSA or computed tomography angiography, CTA) from January 2017 to January 2023. Three patient groups were ultimately included: ICAS-related LVOS, atrial fibrillation (AF)-related LVOS, and symptomatic ICAS (sICAS) without LVO. The classification process involved two neurologists, with a third expert making the final decision when needed (Supplementary Figure S1).

**Figure 1:**
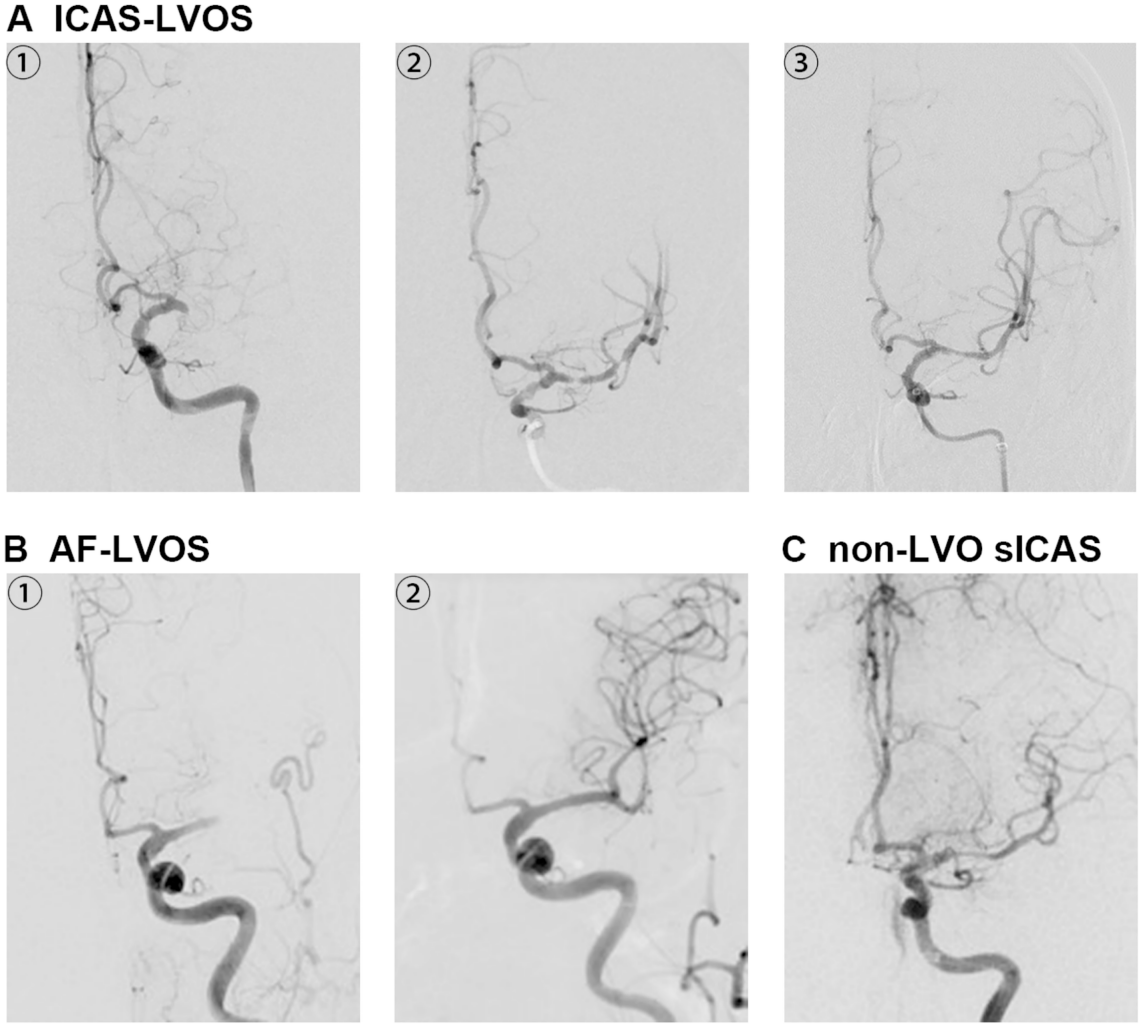
The digital subtraction angiography images of three different groups. **(A)** A1-3 were from a patient of ICAS-LVO. A1 was the initial angiography with the occlusion of the left middle cerebral artery. A2 was the image after mechanical thrombectomy by the stent retriever with residual severe stenosis. A3 was the image after ballon dilating and stent implanting with the stenosis improved. **(B)** B1-2 were from a patient of AF-LVO with a history of atrial fibrillation of more than a decade and irregularly application of anticoagulation before the onset. B1 was the initial angiography with the occlusion of the left middle cerebral artery. B2 was the image after mechanical thrombectomy without any stenosis left. **(C)** C was the initial angiography from a patient of sICAS without the occlusion.

Patients meeting all the following criteria were included in ICAS-LVOS group, based on the definition proposed by European Stroke Organization (6): ① adult AIS patients verified intracranial LVO by DSA and undergoing thrombectomy; ② residual fixed stenosis that measured >50% after several passes of thrombectomy devices or the implantation of a stent; ③ TOAST classification indicating large-artery atherosclerosis. Additionally, characteristic features such as evidence of hypoperfusion or watershed infarction, truncal-type occlusion and early reocclusion aided in patient classification. We excluded patients with evidence of AF to prevent confounding factors (Figure 1A).

Patients meeting all the following criteria were included in AF-LVOS group: ① adult AIS patients verified intracranial LVO by DSA and undergoing thrombectomy; ② no residual stenosis after thrombectomy; ③ diagnosis of AF by electrocardiogram in medical history, at hopsital or during follow-up; ④ TOAST classification indicating cardiac embolism (Figure 1B). To reduce confounding heterogeneity from other, less common cardioembolic sources with distinct pathophysiological mechanisms (e.g., valve replacement, left ventricular thrombus), this study specifically focused on AF as the most prevalent and etiologically homogeneous embolic origin, thereby facilitating a more precise comparison with the ICAS-LVOS group.

Patients meeting all the following criteria were included in sICAS group: ① adult patients with AIS attributed to 50–99% stenosis of main intracranial arteries assessed by CTA or DSA when they arrived at the emergency; ② a new, acute cerebral infarction attributable to the qualifying stenosis confirmed on diffusion-weighted imaging (DWI) by magnetic resonance (MR);. ③ TOAST classification indicating large-artery atherosclerosis. Patients with AF or stenosis resulting from other causes were also excluded (Figure 1C).

### Data collection

For all participants across the three groups, we gathered baseline clinical data encompassing demographics, risk factors, onset-to-door time, NIH Stroke Scale (NIHSS) scores, and blood pressure readings, upon admission. All laboratory tests were performed on blood samples collected at emergency department admission as part of standardized protocol in our center, prior to any therapeutic intervention such as IVT or EVT. Additionally, for ICAS-related LVOS patients, we examined surgical records to ascertain whether specific thrombi were retrieved using thrombectomy devices, serving as a measure of thrombus burden. We also documented the number of passes made with stent retrievers during the procedure and noted any medical therapies administered during hospitalization, including IVT.

### The validation cohort

To further validate the preliminary findings, we established a validation cohort comprising consecutive LVOS patients immediately following the derivation cohort from February 2023 to December 2024. This cohort was sourced from the same prospectively maintained registry and strictly adhered to the identical inclusion and exclusion criteria as the derivation cohort. The purpose was to provide an enternal temporal validation for distinguishing value of the observations in ICAS-LVOS patients (Supplementary Figure S2).

### Statistical analyses

The baseline characteristics and laboratory results were presented as frequencies (percentage) for categorical variables, mean ± standard deviation (SD) for continuous variables with a normal distribution, and median (interquartile range, IQR) for continuous variables with a non-normal distribution. Differences among subgroups for categorical variables were analyzed using Pearson’s chi-square tests or Fisher’s exact tests, while Mann–Whitney tests were employed for continuous variables, as appropriate. Logistic multivariate regression modeling was utilized to identify critical characteristics of ICAS-LVOS compared to AF-LVOS or sICAS. Continuous variables demonstrating significant nonlinearity were log-transformed. Only factors exhibiting significance at *p* < 0.1 in univariate analyses were included in the multivariate analysis. Additionly, interactions analyses were tested between observed inter-group differences and sex in multivariate models. The receiver operating characteristic (ROC) curve was utilized to calculate the pre-diagnostic role and the cut-off value. All tests were two-tailed, with *p* < 0.05 considered statistically significant. All statistical analyses were performed using SPSS version 26.0.

## Results

After screening and exclusions, the final analysis was comprised 70 patients with ICAS-LVOS, 78 patients with AF-LVOS and 131 with sICAS from January 2017 to January 2023. Among the 148 LVOS patients, the mean age was 68.77 ± 12.04 years, with 95 out of 148 (64.2%) being male. In the 201 ICAS patients, the mean age was 64.16 ± 11.97 years, with 152 out of 201 (75.6%) being male, which was consistent with major ICAS trials.

### Comparison in LVOS patients

To investigate the unique characteristics of ICAS-LVOS, we compared them with non-valvular AF-LVOS, another common cause of LVOS with good internal homogeneity. It was found that ICAS-LVOS patients were more likely to be males, younger, had higher body mass index (BMI), were more likely to be smokers, had longer onset-to-door time, lower NIHSS scores, and had a higher prevalence of infarcts in the posterior circulation compared to AF-LVOS. Regarding laboratory results, ICAS-LVOS patients had higher white blood cell (WBC) counts, lower international normalized ratio (INR), higher fibrinogen levels, lower D-dimer levels, higher cholesterol levels and higher triglyceride levels. After logistic multivariate regression analysis, younger age [odds ratio (OR): 0.898; 95%CI: 0.831–0.971, *p* = 0.007], previous stroke (OR: 6.672, 95%CI: 1.183–37.620; *p* = 0.031), posterior circulation (OR: 30.299, 95%CI: 2.157–425.554; *p* = 0.011), higher levels of fibrinogen (OR: 3.421, 95%CI: 1.428–8.194; *p* = 0.006) were independently associated with ICAS as underlying etiology of LVOS (Table 1). Interaction analyses revealed no significant effect modificated by sex (all p for interaction≥0.05), indicating the pattern of differences observed in this study was consistent in both men and women (Supplementary Table S1).

**Table 1:** Baseline clinical characteristics and lab results of ICAS-LVOS and AF-LVOS patients.

Variables	ICAS-LVO (*n* = 70)	AF-LVO (*n* = 78)	*p-*Value	Multivariate analysis	
				OR (95%CI)	*p*-value
Sex, male, *n* (%)	54 (77.1)	41 (52.6)	**0.002**	1.166 (0.244–5.573)	0.847
Age, years, median (IQR)	65.5 (55.0–73.0)	75.0 (69.0–79.0)	**<0.001**	0.898 (0.831–0.971)	**0.007**
BMI, median (IQR)	25.1 (23.7–28.3)	23.7 (22.2–26.0)	**0.010**	1.068 (0.893–1.278)	0.46
Risk factors, *n* (%)					
Previous stroke	18 (25.7)	11 (14.1)	0.076	6.672 (1.183–37.620)	**0.031**
Diabetes Mellitus	19 (27.1)	17 (21.8)	0.449		
Hypertension	50 (71.4)	51 (65.4)	0.430		
Coronary artery diseases	5 (7.1)	13 (16.7)	0.077	2.887 (0.386–21.612)	0.302
Smoke	34 (48.6)	20 (25.6)	**0.004**	0.461 (0.089–2.391)	0.356
Status at admission, median (IQR)					
OTD time, hours	4.9 (2.0–9.4)	2.9 (1.1–6.0)	**0.039**	1.065 (0.934–1.215)	0.344
NIHSS at admission	12.0 (9.5–19.0)	17.0 (14.0–21.0)	**0.002**		
SBP, mmHg	145.5 (130.0–169.0)	144.0 (134.0–163.0)	0.905		
DBP, mmHg	80.5 (73.0–90.0)	82.0 (72.0–89.0)	0.980		
Location, *n* (%)			**0.001**	30.299 (2.157–425.554)	**0.011**
Anterior circulation	48 (68.6)	71 (91.0)			
Posterior circulation	22 (31.4)	7 (9.0)			
Previous anticoagulants, *n* (%)	0 (0)	26 (33.3)	**<0.001**	0.000 (0.000-∞)	0.997
Lab results, median (IQR)					
Blood glucose, mmol/L	6.9 (5.9–10.5)	7.1 (6.4–8.5)	0.991		
WBC, *10^9/L	9.1 (7.4–11.9)	7.7 (6.4–9.9)	**0.013**	1.004 (0.840–1.199)	0.969
INR	0.98 (0.94–1.02)	1.03 (0.98–1.10)	**<0.001**	0.000 (0.000–59.237)	0.167
Fibrinogen, g/L	3.2 (2.6–4.0)	2.7 (2.3–3.3)	**0.001**	3.421 (1.428–8.194)	**0.006**
D-dimer, mg/L	0.42 (0.28–0.94)	1.02 (0.50–2.62)	**<0.001**	0.346 (0.077–1.556)	0.167
LDL-C, mmol/L	2.5 (1.8–3.2)	2.4 (1.9–3.0)	0.814		
Cholesterol, mmol/L	4.6 (3.7–5.3)	4.2 (3.3–4.7)	**0.041**	1.030 (0.519–2.043)	0.933
Triglyceride, mmol/L	1.3 (0.9–1.9)	1.0 (0.6–1.4)	**<0.001**	2.164 (0.796–5.886)	0.131
Homocysteine, μmol/L	13.4 (10.7–18.2)	13.3 (10.2–18.1)	0.789		
Intravenous thrombolysis	28 (40.0)	28 (35.9)	0.607		

ICAS, Intracranial atherosclerotic stenosis; AF, atrial fibrillation; LVOS, Large artery occlusion stroke; IQR, Interquartile range; BMI, Body mass index; OTD, Onset-to-door; NIHSS, NIH stroke scale; SBP, Systolic blood pressure; DBP, Diastolic blood pressure; WBC, White blood cells; INR, International Normalized Ratio; LDL-C, Low-density Lipoprotein Cholesterol.Bold values indicate statistically significant differences (*p* <0.05).

**Figure 2:**
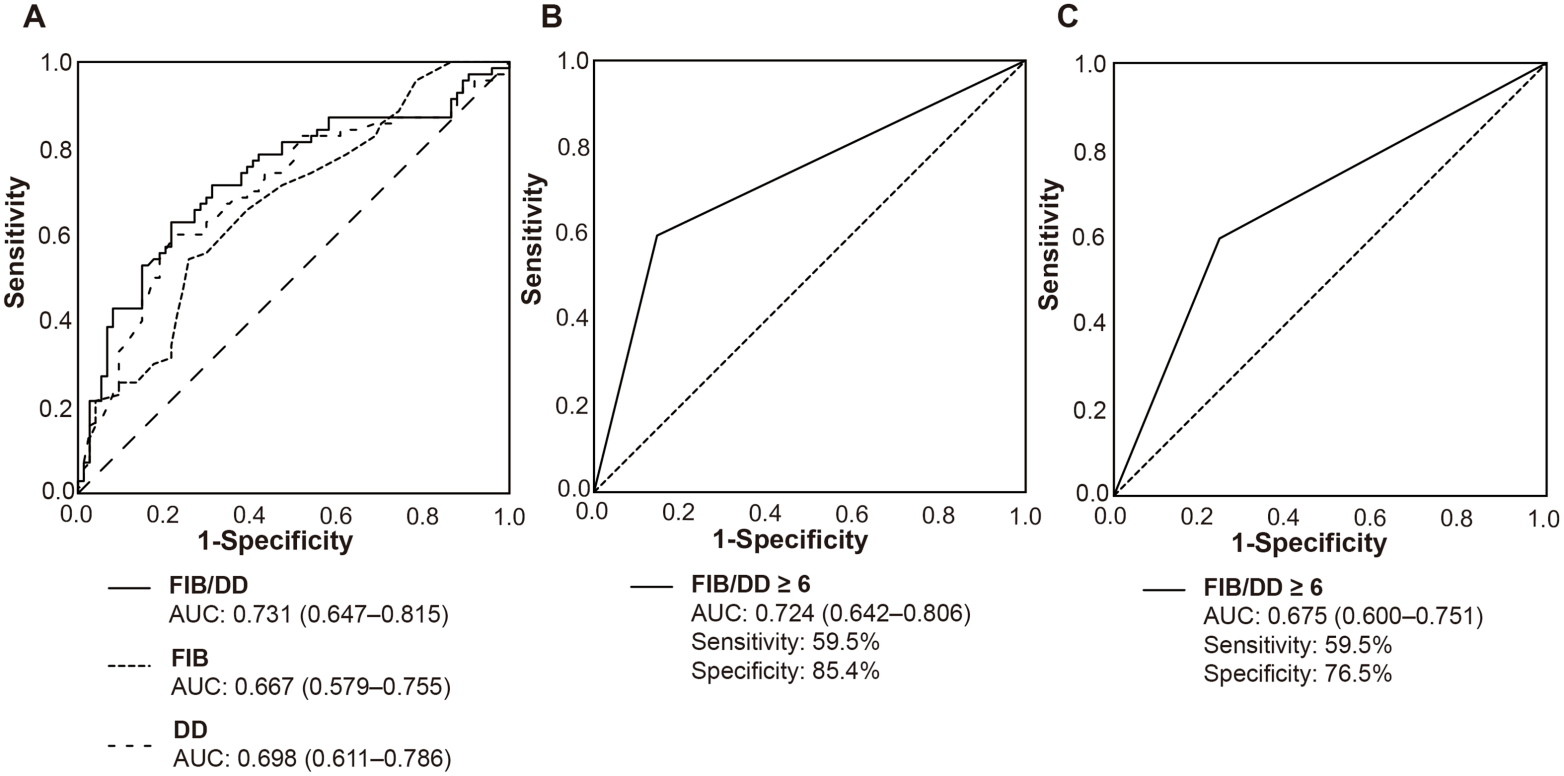
The receiver operating characteristic curves of FIB/DD. **(A)** The derivation cohort. **(B)** The validation cohort with 74 ICAS-LVOS patients and 82 AF-LVOS patients. **(C)** The validation cohort with 74 ICAS-LVOS patients and 176 LVOS patients of all other causes except ICAS-LVOS. Abbreviations: FIB: fibrinogen; D-D: D-dimer; AUC: area under the curve.

Given that fibrinogen serves as a substrate while D-dimer as a degradation product in thrombosis, we therefore assessed the discriminative capacity of fibrinogen, D-dimer, and their ratio (FIB/DD) in identifying ICAS-LVOS from AF-LVOS patients. ROC analysis confirmed that the FIB/DD ratio achieved the highest AUC (0.731, 95% CI, 0.647–0.815) compared to isolated fibrinogen (AUC = 0.667) or D-dimer (AUC = 0.698) (Figure 2A). The option cutoff value of FIB/DD was 6 in the derivation cohort with the sensitivity of 62.9% and specificity of 78.4%. In the validation cohort with 74 ICAS-LVOS patients, 82 AF-LVOS patients, 24 LVOS patients attributed to extracranial atherosclerosis, 10 valve replacement, 3 infective endocarditis, 4 patent foreman oval, 7 other cardiac embolism and 46 other reason or undetermined reason from February 2023 to December 2024, the pre-diagnostic role of FIB/DD ≥ 6 was still reliable, showing an AUC value of 0.724 (95% CI, 0.642–0.806) for differentiating ICAS-LVOS and AF-LVOS and 0.675 (95% CI, 0.600–0.751) for predicting ICAS-LVOS from all LVOS patients, with high specificity (Figures 2B,C).

### Comparison in ICAS patients

To further elucidate the critical factors promoting the in-situ thrombosis and occlusion of large artery in ICAS patients, we compared ICAS-LVOS patients with ICAS-AIS without LVOS. Larger BMI, higher NIHSS scores, higher WBC counts, higher fibrinogen levels were found in ICAS-LVOS patients compared to sICAS (ICAS-non-LVOS). Logistic multivariate regression revealed that higher levels of BMI (OR: 1.176, 95%CI: 1.059–1.306; *p* = 0.002), WBC (OR: 1.234, 95%CI: 1.083–1.406; *p* = 0.002), and fibrinogen levels (OR: 1.600, 95%CI: 1.049–2.440; *p* = 0.029) were significantly associated with ICAS-LVOS. (Table 2).

**Table 2:** Baseline clinical characteristics and lab results of ICAS-LVOS and sICAS patients.

Variables	ICAS-LVOS (*n* = 70)	sICAS (*n* = 131)	*p-*value	Multivariate analysis	
				OR (95% CI)	*p*-value
Sex, male, *n* (%)	54 (77.1)	98 (74.8)	0.714		
Age, years, median (IQR)	65.5 (55.0–73.0)	65.0 (57.0–73.0)	0.602		
BMI, median (IQR) ^a^	25.1 (23.7–28.3)	23.9 (22.3–26.2)	**0.004**	1.176 (1.059–1.306)	**0.002**
Risk factors, *n* (%)					
Previous stroke	18 (25.7)	29 (22.1)	0.568		
Diabetes Mellitus	19 (27.1)	53 (40.5)	0.061	0.530 (0.253–1.114)	0.094
Hypertension	50 (71.4)	105 (80.2)	0.161		
Coronary artery diseases	5 (7.1)	8 (6.1)	>0.99		
Smoke	34 (48.6)	63 (48.1)	0.948		
Status at admission, median (IQR)					
OTD time, hours	4.9 (2.0–9.4)	4.2 (2.1–10.4)	0.756		
NIHSS at admission	12.0 (9.5–19.0)	4.0 (2.0–9.0)	**<0.001**		
SBP, mmHg	145.5 (130.0–169.0)	153.0 (138.0–174.0)	0.082	0.987 (0.973–1.001)	0.069
DBP, mmHg	80.5 (73.0–90.0)	84.0 (77.0–92.5)	0.103		
Anterior circulation, *n* (%)	48 (68.6)	94 (71.8)	0.637		
Lab results, median (IQR)					
Blood glucose, mmol/L	6.9 (5.9–10.5)	7.0 (5.8–9.5)	0.655		
WBC, *10^9/L	9.1 (7.4–11.9)	7.6(6.2–9.5)	**0.001**	1.234 (1.083–1.406)	**0.002**
INR	0.98 (0.94–1.02)	0.96 (0.92–1 0.00)	0.054	17.388 (0.204–1479.3)	0.208
Fibrinogen, g/L	3.2 (2.6–4.0)	2.9 (2.5–3.4)	**0.025**	1.600 (1.049–2.440)	**0.029**
D-dimer, mg/L ^b^	0.42 (0.28–0.94)	0.40 (0.22–0.89)	0.382		
LDL-C, mmol/L ^c^	2.5 (1.8–3.2)	2.8 (2.2–3.2)	0.126		
Cholesterol, mmol/L	4.6 (3.7–5.3)	4.3 (3.7–5.2)	0.699		
Triglyceride, mmol/L	1.3 (0.9–1.9)	1.4 (1.0–2.0)	0.134		
Homocysteine, μmol/L ^d^	13.4 (10.7–18.2)	12.8 (10.1–16.8)	0.789		
Intravenous thrombolysis	28 (40.0)	42 (32.1)	0.260		

sICAS, Symptomatic Intracranial atherosclerotic stenosis.Bold values indicate statistically significant differences (*p* <0.05).

### Thrombus burden in ICAS-LVOS patients

Moreover, to explore the role of fibrinogen in the thrombosis of ICAS-LVOS, we classified these patients into two subgroups: those with and without thrombus extracted by thrombectomy devices such as stent retrievers or aspiration catheters, reflecting the burden level of thrombus. Comparisons were made between the two subgroups, indicating that the group with thrombus extracted had more hypertension, less infarcts in the anterior circulation, higher WBC counts, higher fibrinogen levels and higher homocysteine levels. Similarly, logistic multivariate analysis indicated that a higher proportion of hypertension (OR: 6.071, 95%CI: 1.200–30.708; *p* = 0.029) and higher levels and fibrinogen (OR: 2.322, 95%CI: 1.015–5.313; *p* = 0.046) (Supplementary Table S2) were associated with a higher burden of thrombus, confirming the essential promoting effect fibrinogen in the thrombosis and occlusion of large vessels in ICAS patients.

### Intravenous thrombolysis in ICAS-LVOS patients

**Figure 3:**
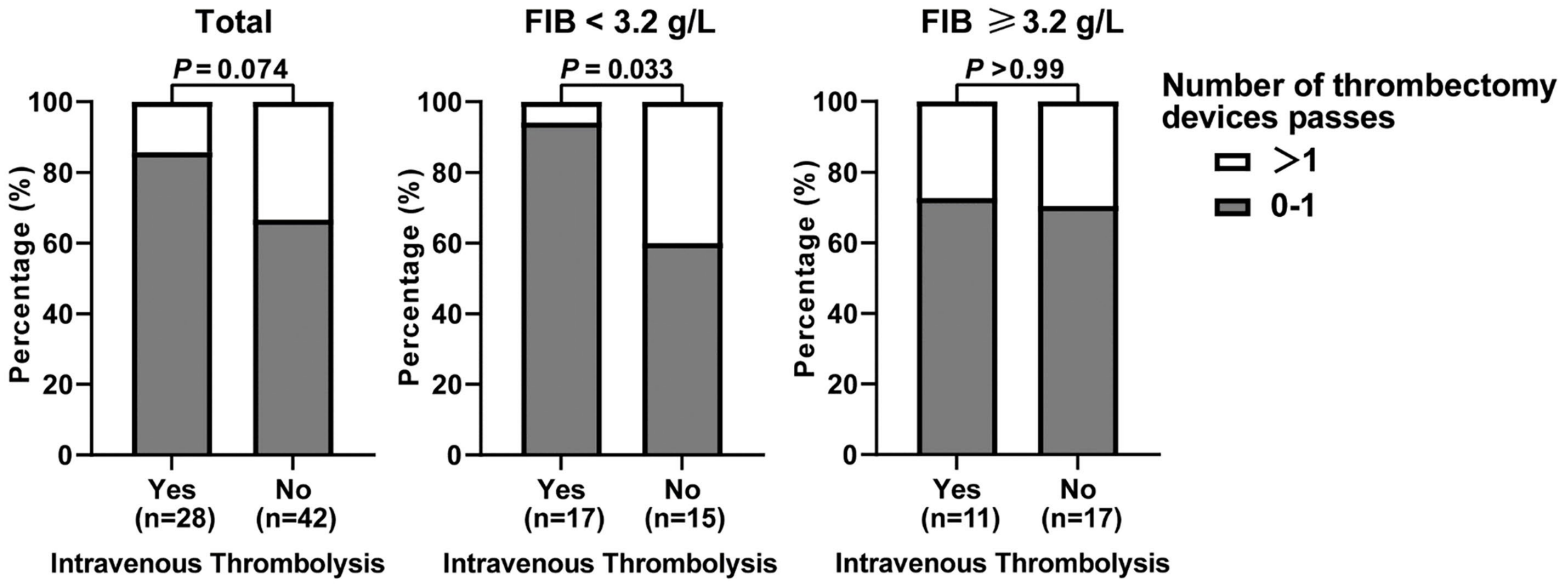
The Association between Thrombolysis and the Number of Mechanical Thrombectomy in ICAS-LVOS Patients with High/Low Burden of Fibrinogen.

On the basis of the association between fibrinogen and thrombus burden, we investigated whether fibrinogen is related to the complexity of thrombectomy. We analyzed the association between fibrinogen levels and the number of thrombectomy device passes. There was no obvious relationship between them (*p* = 0.211). However, in patients who received IVT, the correlation was significant. IVT patients with higher level of fibrinogen underwent more thrombectomy trials (*p* = 0.002), suggesting a potential benefit of IVT preceding MT in ICAS-LVOS patients (Supplementary Figure S3). Additionally, we compared the number of thrombectomy device passes between patients with and without IVT. We observed a trend where more patients who received IVT underwent 0–1 pass of stent retrievers for thrombectomy compared to those without IVT (86% vs. 67%, *p* = 0.074). This trend was particularly notable in patients with lower levels of fibrinogen (fibrinogen <3.2 g/L, which was the median cutoff), where the difference in the reduction of thrombectomy trials with IVT was more significant (94% vs. 60%, *p* = 0.033; Figure 3).

## Discussion

ICAS-LVOS represents a newly recognized condition emerging alongside the widespread use of MT in patients with LVOS (4). Fewer investigations delved into the underlying pathology, particularly why only a subset of patients with ICAS developed LVOS. In our study, we addressed this gap by uncovering the pivotal role of fibrinogen in thrombosis and LVOS occurrence in ICAS patients in real-world practice. Initially, we compared the clinical characteristics and lab results of ICAS-LVOS with AF-LVOS patients and found higher fibrinogen levels significantly associated with ICAS-LVOS. Subsequently, we contrasted ICAS-LVOS with non-LVOS ICAS patients, confirming fibrinogen’s crucial contribution to LVO-associated thrombosis in ICAS. Our analysis of ICAS-LVOS patients with and without extracted thrombi revealed a strong correlation between fibrinogen levels and extensive clot formation. Finally, we investigated the interplay among fibrinogen, IVT, and thrombectomy complexity, discovering that IVT could reduce the number of thrombectomy device passes, particularly in patients with lower fibrinogen levels.

Previous study had figured out the distinct comorbidities between embolism-LVOS and ICAS-LVOS patients (7, 8). In our study, we described the unique characteristics of age, times of stroke, position and distinct levels of fibrinogen in etiologically different patients. In the similar process of thrombosis and LVOS, the reason why the level of fibrinogen was elevated in ICAS-LVOS patients remained uncertain. Retrospectively, numerous studies have identified hyperfibrinogenemia as an independent risk factor for ASCVD and stroke (9, 10). Within the stroke domain, fibrinogen has been associated with etiology classification, clot burden and clinical outcome (11–15). A study has indicated increased fibrinogen in both LAA and CE strokes, with the former accompanied by inflammatory profiles like C-reactive proteins, while the latter by prothrombotic profiles like D-dimers (12), reminding inflammation-driven coagulation in the former and coagulation-driven inflammation in the latter (16). This emphasized the heterogeneous nature of LVOS thrombosis and the need for further understanding and tailored therapeutic approaches.

In our continued efforts to elucidate which subset of ICAS patients would develop LVOS, we conducted a secondary comparison and identified correlations between BMI, WBC counts, and fibrinogen levels in ICAS-associated LVOS compared to non-LVO solitary ICAS patients. It is evident that WBC represents an acute inflammatory response following stroke and is notably higher in LVOS patients (17). Conversely, the influences of BMI and fibrinogen levels can be attributed to the degree of inflammation, particularly chronic aseptic inflammation, given their measurement as stable indicators over a relatively longer period. Fibrinogen, known as coagulation factor I, is converted to fibrin by thrombin, participating in the coagulation cascade, and typically considered as an acute-phase reactant synthesized by the liver under the activation of inflammatory mediator like interleukin-6 (9, 16). However, it was demonstrated that the level of fibrinogen remained stable in the first 24 h after stroke onset, and peaked until over 3 days or longer (14, 15, 18). Therefore, in our study, fibrinogen levels were measured at admission without any treatment, suggesting that they reflect the chronic inflammatory state preceding thrombosis. This underscores the intricate interplay between inflammation, atherosclerosis, and thrombosis, necessitating a comprehensive understanding for effective therapeutic interventions.

As previously discussed, the characteristic of ICAS-LVOS was inflammation-driven coagulation. Consequently, it is plausible to hypothesize that individuals with higher levels of inflammatory could be predisposed to unstable plaque rupture and subsequent LVOS. This hypothesis finds parallels in the realm of acute coronary syndrome (ACS), where those ruptured fibrous cap (RFC) exhibits markedly elevated levels of interleukin-6 and other inflammatory proteome expression compared with intact fibrous cap (IFC) (19). Interestingly, RFC-associated ACS involves plaque rupture, while IFC-associated ACS is attributed to plaque erosion, leading to different clot characteristics (20). A case report detailing the occlusion of the basilar artery in ICAS- LVOS has indicated a similar mechanism of IFC. Regretfully, the widespread prevalence of this phenomenon in ICAS-associated LVOS remains unknown (21). Future research endeavors should prioritize elucidating this aspect, as it holds implications for tailored therapeutic strategies. It has been proved that plaques exhibiting IFC may benefit more from anti-thrombotic therapies (22).

Moreover, our study identified hypertension as a key factor associated with greater thrombus burden in ICAS-LVOS. On one hand, hypertension fosters a systemic pro-inflammatory state, which upregulates fibrinogen synthesis (23). The environment of elevated fibrinogen levels have been demonstrated to confer mechanical stability to clots, increasing their rigidity and impairing permeability (24). It correlated with the poor clinical outcomes following IVT in higher fibrinogen levels (14, 15). On the other hand, hypertension may directly promote a pro-thrombotic state, contributing to the formation of clots with a more compact fibrin network and a predisposition toward hypofibrinolysis (25). These unfavorably altered fibrin clot properties can contribute to a higher thrombus burden. Therefore, rigorous management of hypertension and elevated fibrinogen levels emerges as a potential therapeutic strategy to mitigate thrombus burden and possibly improve EVT outcomes in ICAS-LVOS.

The challenges of early and recurrent reocclusion persist in the endovascular treatment. Several retrospective studies have revealed the potential rescue therapies (26). Nevertheless, the utility of pretreatment before thrombectomy remains uncertain. While direct thrombectomy has not proven inferior to bridging thrombectomy with IVT (27), Asian patients, who exhibit a higher prevalence of ICAS-LVOS, may derive greater benefit from bridging therapy (28). Latest study has demonstrated the prevailage of IVT with tenecteplase before thrombectomy (29). Our study also observed a signal that the reduction in thrombectomy device passes associated with IVT appeared more pronounced in the subgroup of ICAS-LVOS patients with lower fibrinogen levels, although it is critical to note that this subgroup analysis was underpowered due to the small sample size. Lower fibrinogen levels might enhance the penetration and efficacy of thrombolytic agents, facilitating early clot lysis and leading to fewer device passes, which aligns with the established role of IVT in promoting early recanalization (27). Conversely, the observed attenuated effect in patients with high fibrinogen levels suggests that fibrinogen might act as a barrier to thrombolysis. This exploratory observation raises the possibility that future therapeutic strategies, such as the use of fibrinogen-lowering agents (e.g., fibrinogenase (30)) or anti-inflammatory therapies, could be investigated to potentially improve outcomes in this specific patient population. However, any such implications are speculative at this stage and require rigorous future validation.

Our study had several limitations. Firstly, classification accuracy relied on diagnostic tests (CTA/DSA) and varied strategies of MT by different interventionalists. Potentially introducing misclassification biases. Secondly, being a cross-sectional study, it lacked longitudinal research on the association, thereby limiting the convincement of the causal link between fibrinogen and ICAS to some extent. Therefore, cohort studies are crucial to fully establish this relationship in the future. Thirdly, the analysis of IVT’s effect on MT in ICAS-LVOS patients was limited by the small number of recruited patients. Adjusting for other confounding factors, such as onset-to-door time and the use of antiplatelet and heparins therapies, was not possible. Lastly, our study primarily focused on fibrinogen in the pathophysiology of ICAS-LVOS, but lifestyle factors, comorbidities, plaque characteristics, and inflammatory levels are also pivotal and should be included in forthcoming studies.

## Conclusion

In summary, this observational study highlights the role of fibrinogen in facilitating thrombosis and occurrence of LVOS in ICAS patients. The higher level of fibrinogen observed in ICAS-LVOS patients compared to AF-LVOS patients suggest two distinct mechanisms of thrombosis and provide a method of prediagnosis. Additionally, elevated fibrinogen levels in ICAS-LVOS patients compared to sICAS-non-LVOS patients indicate fibrinogen’s contribution to thrombosis in this subgroup. Furthermore, fibrinogen levels correlate with thrombus burden, suggesting that IVT may alleviate the challenges of thrombectomy in patients with lower fibrinogen burden. These findings underscore the importance of fibrinogen as a potential target in the assessment and treatment strategies for ICAS-LVOS. Therapeutic interventions aimed at reducing fibrinogen levels, such as anti-inflammatory approaches to inhibit synthesis or fibrinogenase to degrade existing fibrinogen, hold promise for future benefits.

## Data Availability

The original contributions presented in the study are included in the article/Supplementary material, further inquiries can be directed to the corresponding authors.
