# Supplementary material for: Higher fibrinogen levels contributes to thrombosis in intracranial atherosclerosis-related large vessel occlusion strokes
Source: Front Neurol. 2026 Mar 4;17:1753192. doi: 10.3389/fneur.2026.1753192 (PMC12996074; doi:10.3389/fneur.2026.1753192)

Higher Fibrinogen levels Contributes to Thrombosis in Intracranial Atherosclerosis-Related Large Vessel Occlusion Strokes

Supplemental Material

Figure S1 Study Flowchart.

Figure S2 Study Flowchart of the Validation Cohort.

Table S1 Interaction Analysis for Sex and the Observed Differences.

Table S2 Baseline Clinical Characteristics and Lab Results of ICAS-LVOS Patients With/Without Thrombus extracted by Thrombectomy.

Figure S3 The Association between Fibrinogen and the Number of Mechanical Thrombectomy in ICAS-LVOS Patients

Figure S1 Study Flowchart.

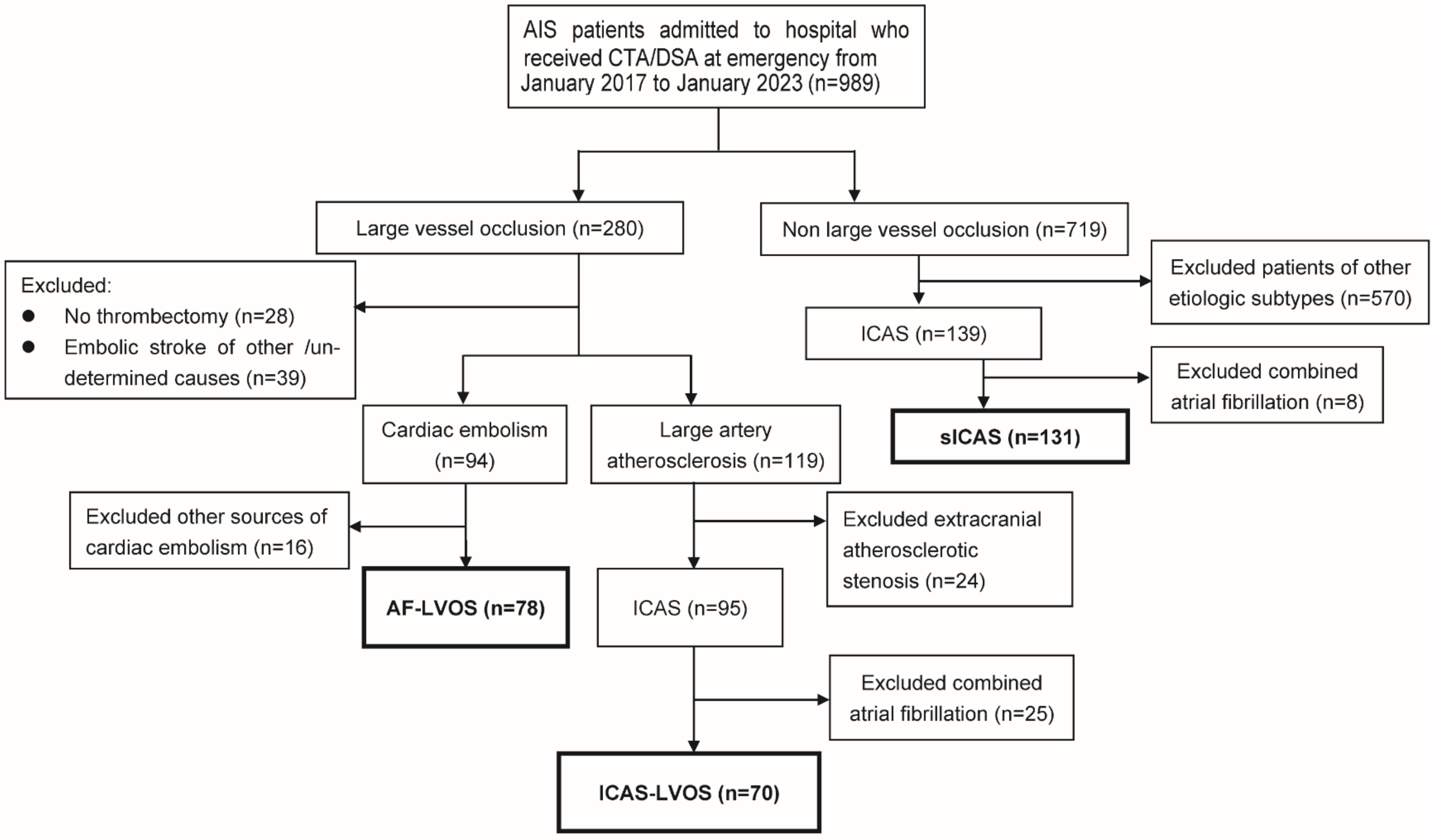

Figure S2 Study Flowchart of the Validation Cohort.

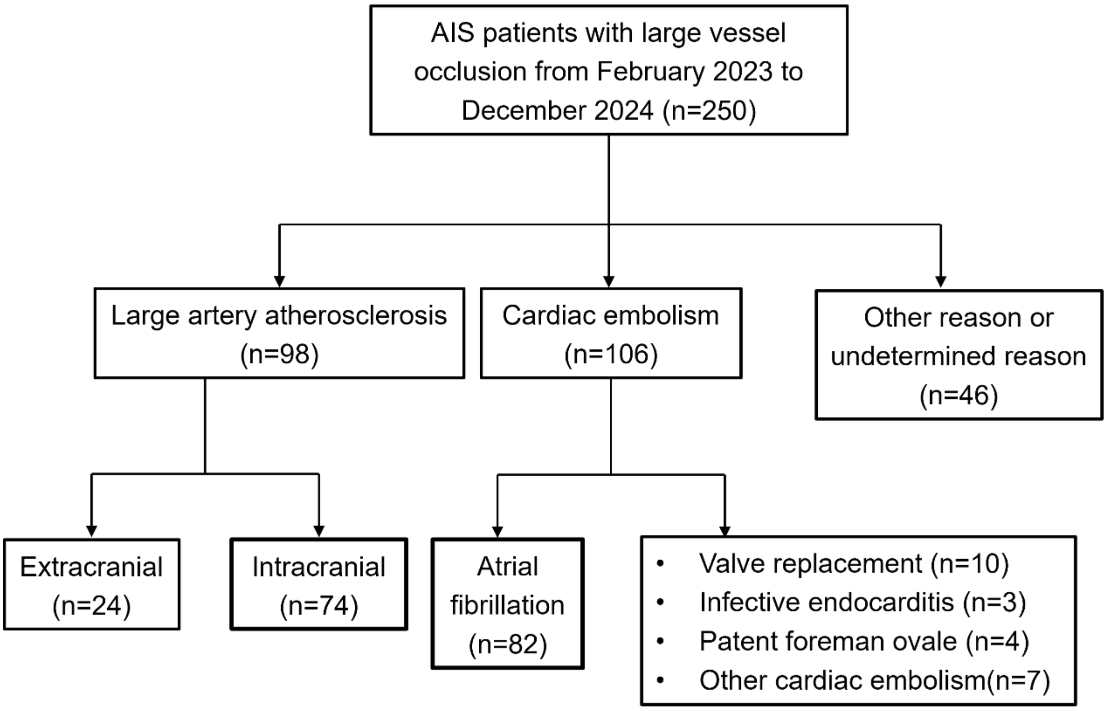

Table S1 Interaction Analysis for Sex and the Observed Differences.

Interaction terms	P for interaction
Sex * Age	0.05
Sex * Previous stroke	0.82
Sex * Location	0.31
Sex * Fibrinogen	0.29

Table S2 Baseline Clinical Characteristics and Lab Results of ICAS-LVOS Patients With/Without Thrombus extracted by Thrombectomy.

	Thrombus (n=32)	Non-thrombus (n=38)	P Value	Multivariate Analysis	
				OR (95%CI)	P-value
Sex, male, n (%)	26 (81.3)	28 (73.7)	0.453		
Age, years, median (IQR)	63.0 (51.0-70.0)	67.0 (56.0-74.0)	0.198		
BMI, median (IQR) [a]	24.8 (23.6-28.1)	25.6 (23.7-28.5)	0.916		
Risk factors, n (%)					
Previous stroke	6 (18.8)	12 (31.6)	0.221		
Diabetes Mellitus	7 (21.9)	12 (31.6)	0.363		
Hypertension	28 (87.5)	22 (57.9)	**0.006**	6.071 (1.200-30.708)	**0.029**
Coronary artery diseases	2 (6.3)	3 (7.9)	>0.99		
Smoke	17 (53.1)	17 (44.7)	0.484		
Status at admission, median (IQR)					
OTD time, hours	5.1 (2.1-9.5)	4.4 (1.8-9.0)	0.520		
NIHSS at admission	13.0 (10.0-25.5)	12.0 (9.0-17.0)	0.250		
SBP, mmHg	151.0 (133.0-176.0)	141.0 (127.0-163.0)	0.325		
DBP, mmHg	84.5 (74.0-96.5)	79.5 (70.0-89.0)	0.181		
Anterior circulation, n (%)	17 (53.1)	31 (81.6)	**0.011**	0.329 (0.075-1.443)	0.141
Intravenous thrombosis	10 (31.3)	18 (47.4)	0.170		
mTICI 2b-3, n (%)	29 (90.6)	32 (84.2)	0.494		
Lab results, median (IQR)					
Blood glucose, mmol/L	7.1 (6.6-9.6)	6.8 (5.7-10.6)	0.468		
WBC, *10^9/L	10.4 (8.2-12.6)	8.1 (7.1-9.9)	**0.005**	1.133 (0.882-1.456)	0.327
INR	0.98 (0.94-1.01)	0.98 (0.93-1.02)	0.972		
Fibrinogen, g/L	3.4 (2.9-4.3)	3.0 (2.6-3.5)	**0.031**	2.322 (1.015-5.313)	**0.046**
D-dimer, mg/L	0.44 (0.29-0.97)	0.39 (0.23-0.80)	0.501		
LDL-C, mmol/L [b]	2.8 (1.6-3.2)	2.5 (1.8-3.1)	0.500		
Cholesterol, mmol/L	4.9 (3.5-5.4)	4.3 (3.9-5.1)	0.541		
Triglyceride, mmol/L	1.2 (0.9-1.9)	1.3 (0.8-2.0)	0.931		
Homocysteine, μmol/L [c]	16.1 (12.6-19.4)	11.7 (9.5-15.2)	**0.026**	1.069 (0.947-1.208)	0.280

Figure S3 The Association between Fibrinogen and the Number of Mechanical Thrombectomy in ICAS-LVOS Patients

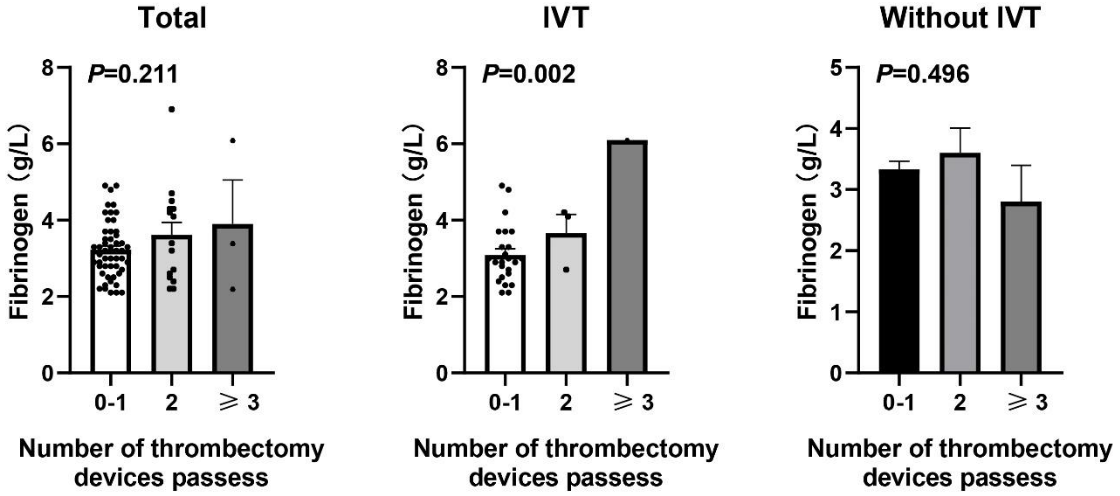

Supplement: Supplementary file 1 [file Data_Sheet_1.pdf]
